# Supplementary material for: Meis1 Controls the Differentiation of Eye Progenitor Cells and the Formation of Posterior Poles during Planarian Regeneration
Source: Int J Mol Sci. 2023 Feb 9;24(4):3505. doi: 10.3390/ijms24043505 (PMC9961902; doi:10.3390/ijms24043505)
Supplement: Supplementary file 1 [file ijms-24-03505-s001.zip › ijms-2067554-supplementary.pdf]

Supplementary Information

Supplementary Information Index

- Supplementary Figure S1
- Supplementary Figure S2
- Supplementary Figure S3
- Supplementary Figure S4
- Supplementary Figure S5
- Supplementary Table S1

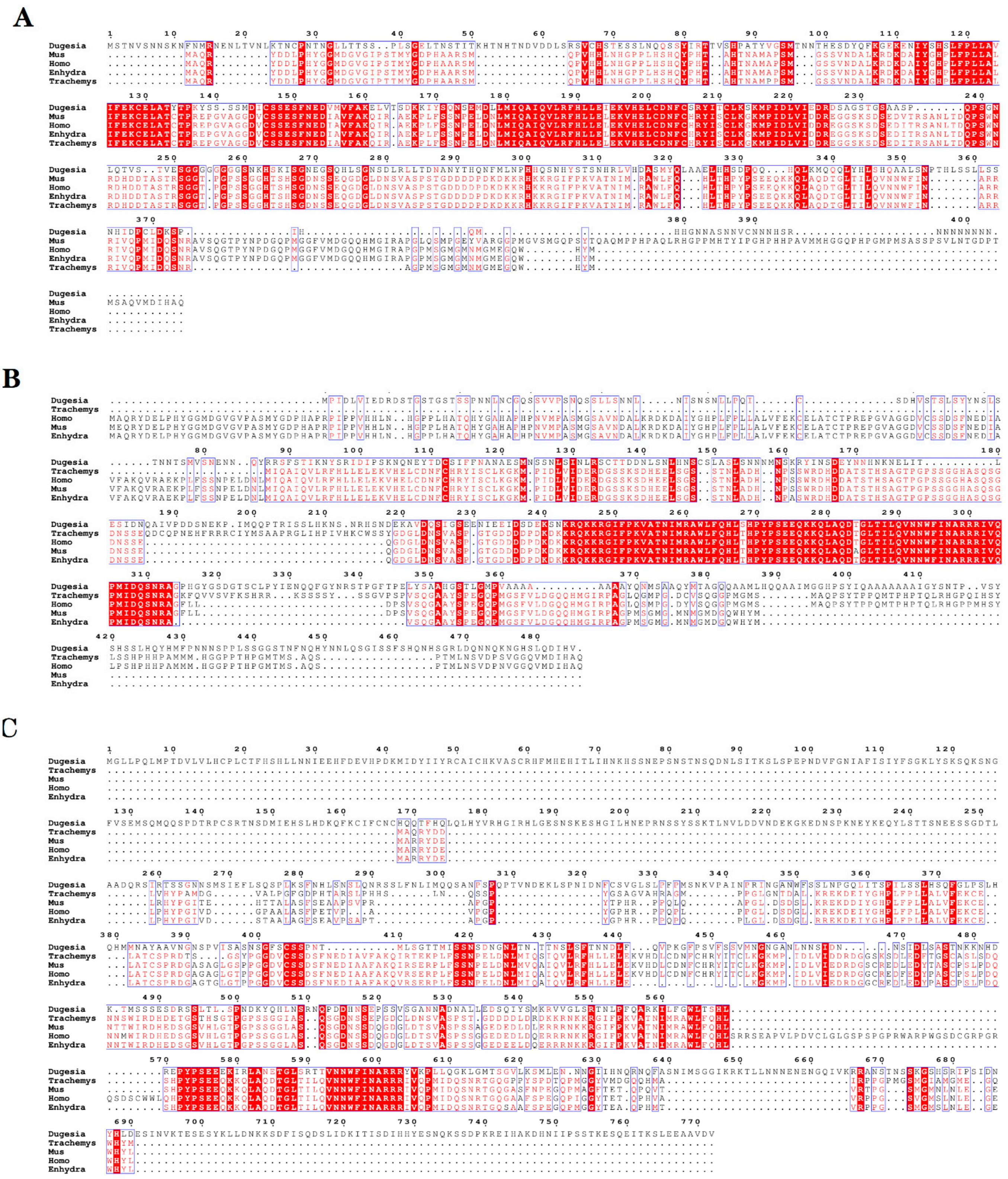

Figure S1. Multiple sequence alignment of *DjMeis1, DjMeis2 and DjMeis3* from *Dugesia japonica*, *Homo sapiens*, *Mus musculus, Trachemys scripta elegans, and Enhydra lutris kenyoni.* (A) *DjMeis1,* (B) *DjMeis2*, (C) *DjMeis3*.Absolutely conserved residues are in red shaded boxes, and highly conserved residues are colored red and are boxed.

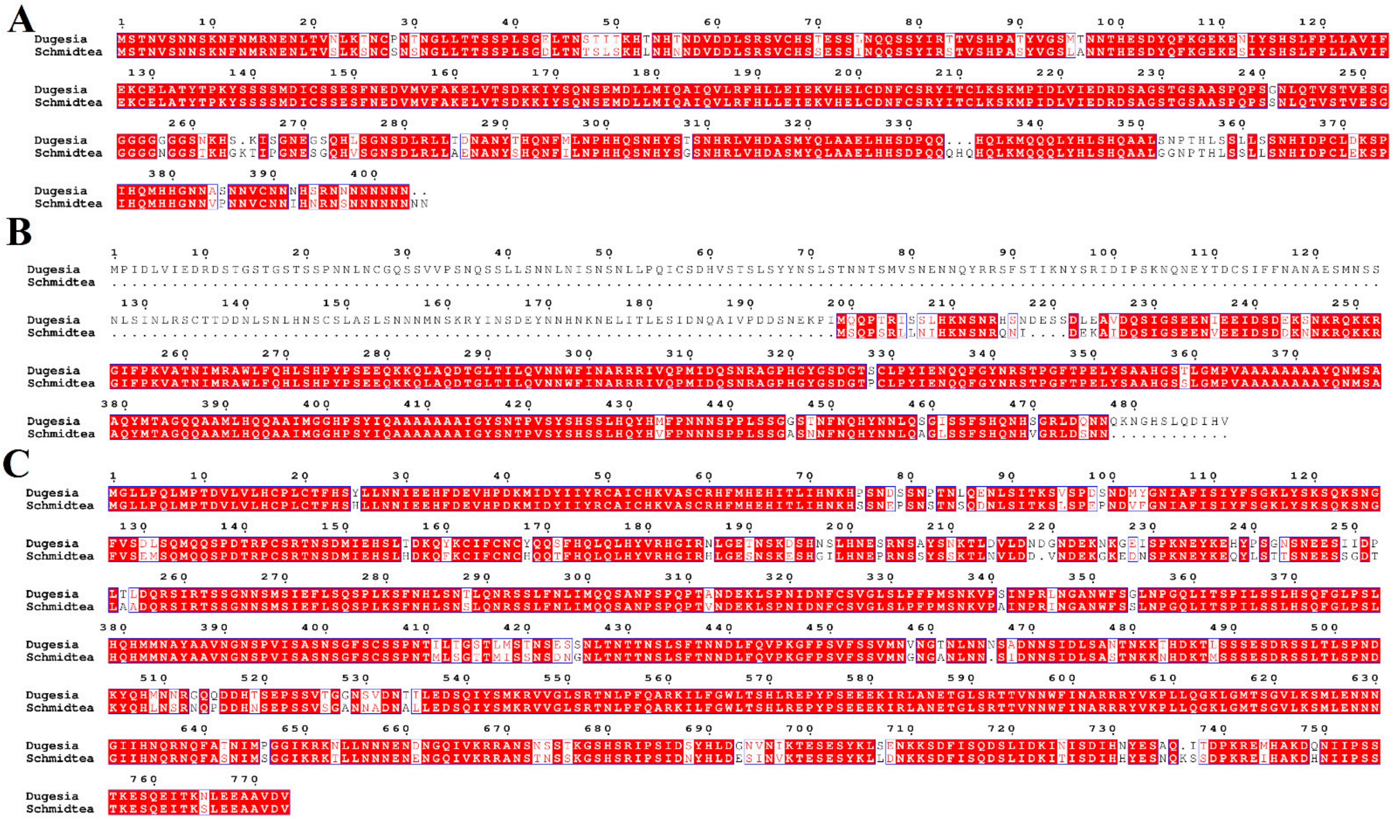

Figure S2 The comparison of Meis family proteins between *Dugesia japonica and Schmidtea mediterranea. (*A) Meis1. (B) Meis2. (C) Meis3. Absolutely conserved residues are in red shaded boxes. and highly conserved residues are colored red and are boxed.

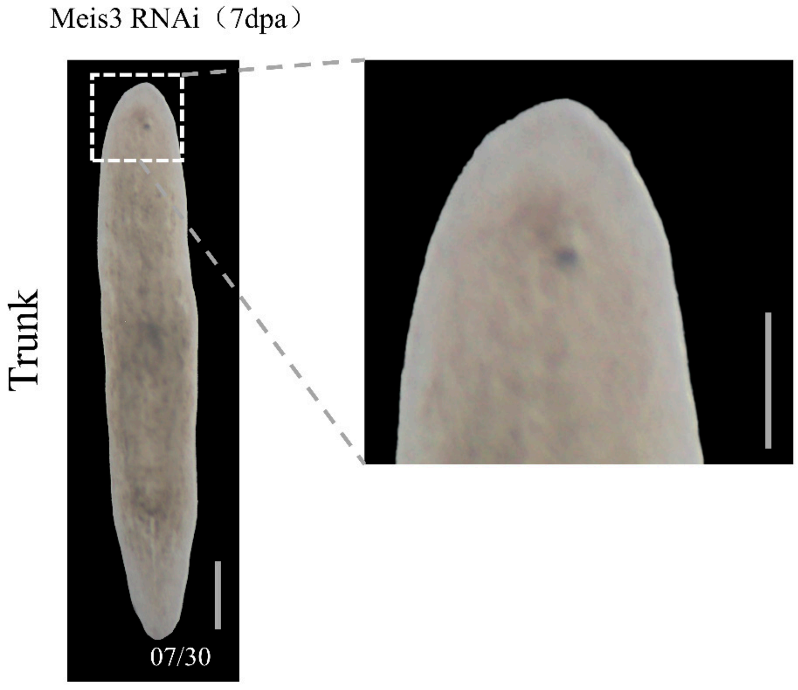

Figure S3 *DjMeis3* RNAi animals (07/30) displayed cyclopes at 7dpa. Scale bars: 400μm.

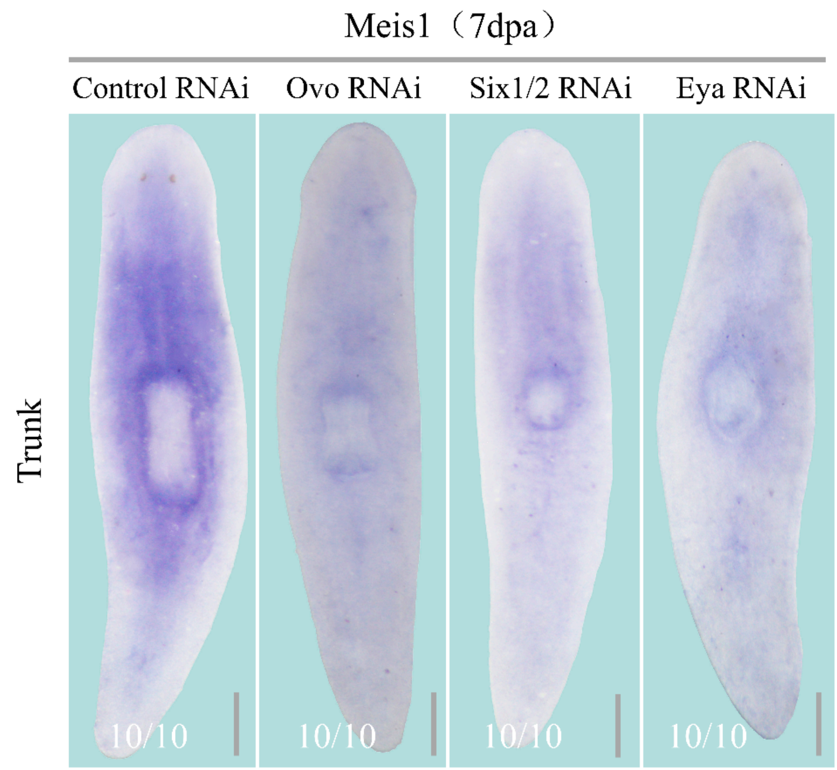

Figure S4 The expression pattern of *DjMeis1* after knockdown of *Djovo*, *Djsix1/2* and *Djeya*, respectively. The expression of *DjMeis1* was suppressed after knockdown of *Djovo*, *Djsix1/2* and *Djeya*. Scale bars: 400μm.

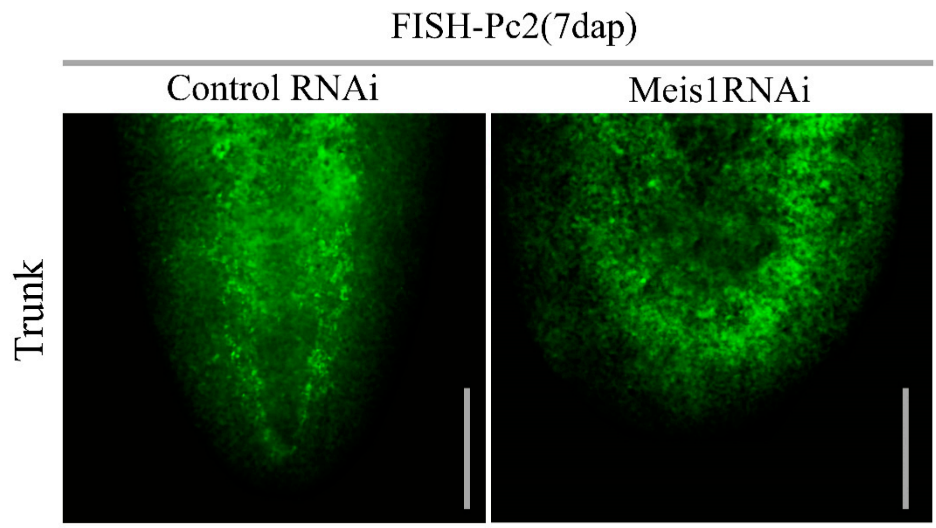

Figure S5 The images of VNC by FISH. *DjMeis1* animals did not properly regenerate VNC but directly connected VNC at wound site. Scale bars: 400μm.

Table S1. PCR primers used in this study

**DjMeis1F**	CATTGAATCAACAGTCGTC
**DjMeis1R**	GATCTGAATGATGGAGTTC
**T7- DjMeis1F**	TAATACGACTCACTATAGGCATTGAATCAACAGTCGTC
**T7- DjMeis1R**	TAATACGACTCACTATAGGGATCTGAATGATGGAGTTC
**DjMeisF**	CTTGAGATCATGTACAACTG
**DjMeisR**	CATAGGTTGAACTATTCGTC
**T7-DjMeisF**	TAATACGACTCACTATAGGCTTGAGATCATGTACAACTG
**T7-DjMeisR**	TAATACGACTCACTATAGGCATAGGTTGAACTATTCGTC
**DjMeis-likeF**	GCAATTGCATTATGTTCGAC
**DjMeis-likeR**	CTGCATAAGCATTCATCATG
**T7-DjMeis-likeF**	TAATACGACTCACTATAGGGCAATTGCATTATGTTCGAC
**T7-DjMeis-likeR**	TAATACGACTCACTATAGGCTGCATAAGCATTCATCATG
**Djβ-cateninF**	CTAACAGTAGTAATTAACAG
**Djβ-cateninR**	CTGAAGCAATTGAAACTATC
**T7-Djβ-cateninF**	TAATACGACTCACTATAGGCTAACAGTAGTAATTAACAG
**T7-Djβ-cateninR**	TAATACGACTCACTATAGGCTGAAGCAATTGAAACTATC
**DjovoF**	GATCCACTAATTCCAGTAC
**DjovoR**	CATGACGTACTATAACTAC
**T7-DjovoF**	TAATACGACTCACTATAGGGATCCACTAATTCCAGTAC
**T7-DjovoR**	TAATACGACTCACTATAGGCATGACGTACTATAACTAC
**Djsix1/2F**	CCGACATCTCAAATTACTTG
**Djsix1/2R**	CGTATACGATTCAAGAATTCTG
**T7-Djsix1/2F**	TAATACGACTCACTATAGGCCGACATCTCAAATTACTTG
**T7-Djsix1/2R**	TAATACGACTCACTATAGGCGTATACGATTCAAGAATTCTG
**DjeyaF**	CTGACCAAGCTCATCAACAAC
**DjeyaR**	GATCTTGTCCATTGTCATCTG
**T7-DjeyaF**	TAATACGACTCACTATAGGCTGACCAAGCTCATCAACAAC
**T7-DjeyaR**	TAATACGACTCACTATAGGGATCTTGTCCATTGTCATCTG
